# Prognostic factors for mortality in patients with bullous pemphigoid: a meta-analysis

**DOI:** 10.1007/s00403-017-1736-1

**Published:** 2017-03-19

**Authors:** Yi-Di Liu, Yan-Hong Wang, Yi-Cong Ye, Wen-Ling Zhao, Li Li

**Affiliations:** 1 Department of Dermatology, Peking Union Medical College Hospital, Peking Union Medical College and Chinese Academy of Medical Science, Beijing, 100730 China; 2 Department of Epidemiology and Bio-statistics, Institute of Basic Medical Sciences, China Academy of Medical Sciences and Peking Union Medical College, Beijing, 100730 China; 3 Department of Cardiology, Peking Union Medical College Hospital, Peking Union Medical College and Chinese Academy of Medical Science, Beijing, 100730 China

**Keywords:** Bullous pemphigoid, Autoimmune blistering disease, Prognosis, Mortality, Survival

## Abstract

Bullous pemphigoid (BP) is a chronic debilitating autoimmune blistering disease that frequently occurs in the elderly population. Previous studies have suggested a high morbidity and mortality associated with BP. However, relatively few studies have investigated prognostic factors of BP mortality, and they showed considerably various results. This meta-analysis aimed to quantitatively assess the association between several potential prognostic factors and risk of mortality in bullous pemphigoid. A comprehensive search was performed using Pubmed, Embase, and Cochrane Library. Cohort studies that assessed prognostic factors of BP mortality were included. Random-effects model was utilized to calculate the pooled hazard ratio (HR). Publication bias was evaluated qualitatively by constructing a funnel plot and quantitatively by conducting Egger’s test. 14 studies were included comprising 2499 patients. Combined HRs suggested that advanced age (HR 1.63, 95% CI 1.34–1.97), presence of circulating antibodies (HR 1.77, 95% CI 1.20–2.62), concomitant dementia (HR 2.01, 95% CI 1.22–3.33), and concomitant stroke (HR 1.86, 95% CI 1.29–2.67) have an unfavorable impact on patient survival. Gender, disease extent, mucosal involvement, and indirect immunofluorescence result were not shown to be linked to mortality by our analysis. This study indicated that BP patients with older age, circulating antibodies, dementia, and stroke are at greater risk of mortality. Clinicians should be aware of this association and utilize this information in patient education and treatment process.

**Electronic supplementary material:**

The online version of this article (doi:10.1007/s00403-017-1736-1) contains supplementary material, which is available to authorized users.

## Introduction

Bullous pemphigoid (BP) is a chronic debilitating autoimmune blistering disease which has a high mortality and morbidity [28]. BP mainly affects the elderly people, causing symptoms, such as widespread tense blistering and itching. Previous studies reported an incidence of BP ranging from 2.5 to 42.8 cases per million per year [2, 11, 14, 23]. BP has been reported to be significantly associated with mortality, with morality rates ranging from 6 to 41% within the first year after diagnosis [1, 3–6, 12, 15, 22, 27, 29, 30, 32]. BP patients also have increased mortality compared with their age and sex-matched controls in the general population [2–4, 12, 14].

However, relatively few studies have investigated prognostic factors of BP mortality, with considerably various results. Old age has been shown to be related to poor outcome in BP in several studies [6, 12, 15, 27], but there are conflicting results on whether old age is an independent risk factor for mortality in BP [3, 21, 22]. In addition, poor general condition and low karnofsky scale [26] have often been associated with increased mortality [1, 15, 21, 29]. Interestingly, factor directly related to BP, such as the severity and extent of skin lesion, does not often predict survival [3, 12, 21, 22].

In addition, BP patients have complex comorbidity profiles, most notably neurological disorders as well as autoimmune, infectious and cardiovascular disorders [28]. Various studies have confirmed a strong association between BP and neurological diseases, particularly dementia, stroke, Parkinson disease, multiple sclerosis, and epilepsy [20]. BP is associated with development of autoantibodies directed against the hemidesmosomal BP autoantigens BPAG1 and BPAG2. BPAG1 has two isoforms: BPAG1-e is mainly expressed in skin, while BPAG1-a is expressed in central nervous system [20]. It is commonly suggested that an immunological cross-reaction between these isoforms could be the pathogenic reason. However, the prognostic value of co-existing neurological disorders as well as other comorbidities for BP mortality is unclear.

In addition, the sample sizes of these studies are often relatively small, leading to limited statistical power to detect true associations. In addition, there is still no systematic and quantitative assessment of published findings on this topic. We, therefore, conducted a meta-analysis to quantitatively assess the association between several potential prognostic factors and risk of mortality in bullous pemphigoid.

## Materials and methods

This meta-analysis was conducted according to the Preferred Reporting Items for Systematic Reviews and Meta-Analyses (PRISMA) Statement protocol [25].

### Search strategy and study selection

We performed an exhaustive search on Pubmed (from 1978 to November 2016), Embase (from 1974 to November 2016) and Cochrane Library (from 1994 to November 2016) to identify articles that studied the prognostic factors for BP mortality. There was no limit on human race, geographic region, language, or publication type. The major terms used included: “bullous pemphigoid”, “mortality”, “fatality”, “survival”, “death”, “prognosis”, and “prognostic”. The following selection criteria were performed on Pubmed as an example:

1. bullous pemphigoid [Title] or bullous pemphigoid [Mesh Terms];
2. mortality or fatality or survival or death or prognosis or prognostic [All Fields];
3. 1 and 2.

Studies eligible for inclusion in this meta-analysis met the following criteria: (a) cohort study design; (b) measure potential prognostic factors for BP mortality; (c) provide sufficient data for calculating the effect size with 95% confidence intervals (CIs); and (d) when the same author’s data obtained from the same or overlapping patients in more than one publication, only the most recent report or the most complete one was selected in the analysis. We excluded case reports, reviews, meta-analyses, and letters. Studies investigating remission or relapse but not mortality of BP were excluded. Childhood bullous pemphigoid was not considered in the present study. Additional studies were identified by a manual search of the references of original studies. The literature search and inclusion of eligible articles was performed independently by two authors (Liu YD and Zhao WL) and disagreements were resolved at each step by consensus.

### Data extraction

All data were extracted independently and crosschecked by two authors (Liu YD and Zhao WL). Data retrieved from the studies included authors, year of publication, patient source, number of patients included in the study, study type, follow-up time, and statistical results regarding the influence of prognostic factors on the risk of mortality. If both 1 year mortality and overall mortality were reported in a study, 1 year mortality was extracted for analysis.

### Quality assessment

Quality assessment of each study was performed independently by the two authors (Liu YD and Zhao WL), using the Newcastle–Ottawa Scale (NOS) (http://www.ohri.ca/programs/clinical_epidemiology/oxford.asp), which graded studies according to the quality of selection, comparability, and outcome of study participants. Studies that achieved seven or more stars on NOS were considered to be of high quality, four to six stars were medium quality, and fewer than four stars were poor quality [24]. Discrepancies were addressed by re-evaluation of the original article and discussion with a third author (Wang YH).

### Statistical methods

For the quantitative aggregation of the survival results, we reported summary measures of effect size using a pooled hazard ratio (HR) with 95% confidence interval. If available, we extracted the risk estimates that underwent multivariate adjustment. For studies in which the HR corresponding to the 95% CI was not given directly, published data from original papers were used to calculate the HR according to the methods described previously [31]. In a few situations where there was not sufficient information for HR estimation, we calculated the relative risk (RR) with 95% CI as a crude substitution of HR. Study heterogeneity was assessed using the Cochran Q test with *P* value for significance set at 0.10 and the *I* ^2^ statistic with values of 25, 50 and 75% considered as low, moderate, and high heterogeneity, respectively [13]. To estimate the pooled HR, random-effects model of DerSimonian and Laird was used [7]. Publication bias was evaluated with funnel plot and Egger’s test (*P* < 0.05 was considered statistically significant) [9]. The statistical analysis was performed by the STATA 14.0 software (StataCorp, College Station, TX, USA).

## Results

### Study characteristics

**Fig. 1:**
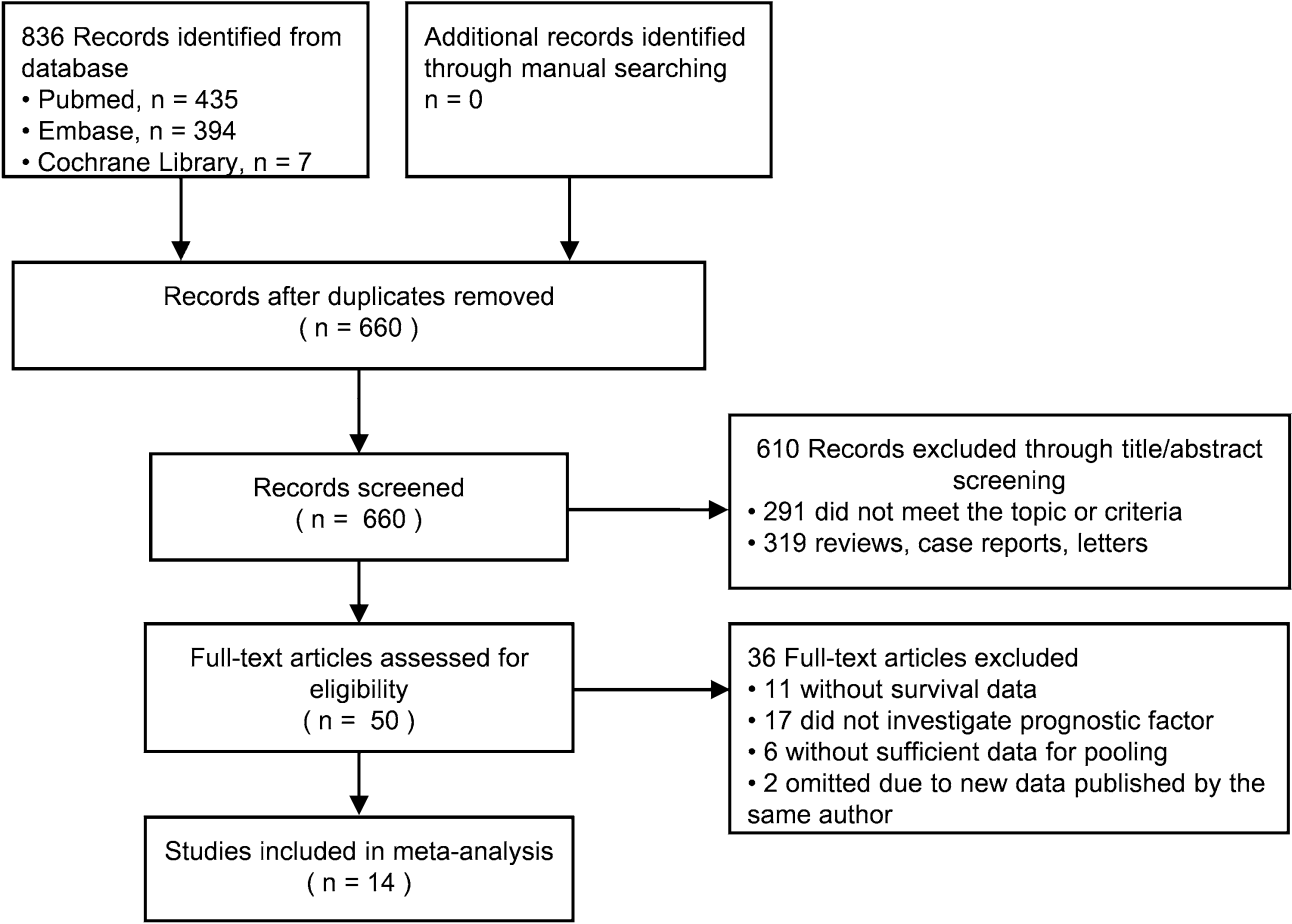
Flowchart of studies included in the meta-analysis

The preliminary literature search returned 660 studies matching the initial search criteria, as shown in Fig. 1. After screening, we identified 14 studies on ten Caucasian and four Asian population samples, including a total of 2499 patients after extracting overlapping patients between studies. The major characteristics of the 14 eligible publications were reported in Table 1. Of the 14 studies, nine were retrospective cohort studies, and five were prospective cohort studies. Based on NOS, 13 studies were considered to be of high quality and one was of medium quality, as shown in Table 2. Most studies were conducted in major centers with an exception of one Swiss study which was population-based [6]. When there was identical or overlapping patient population between the 14 studies, we only included the study with the more complete population in the investigation of a specific prognostic factor of interest [1, 15, 16, 29].

**Table 1:** Main characteristics of the eligible studies

First author	Year	Country	Sample size	Study type	Type of care setting	HR estimation	Follow up time	Study quality
Lee	2014	Korea	168	Retrospective	Tertiary center	1-year mortality	2.19 years^a^	8
Cai	2014	Singapore	359	Retrospective	Tertiary center	Overall mortality	3 years	8
Gual	2014	Spain	101	Retrospective	Tertiary center	1-year mortality	1 year	9
Li	2013	China	140	Retrospective	Inpatient	Overall mortality	3 years^a^	8
Zhang	2013	China	94	Retrospective	Inpatient	1-year mortality	1 year	9
Cortés	2012	Switzerland	60	Retrospective	Inpatient	Overall mortality	1 to 5 years	8
Cortés	2011	Switzerland	115	Prospective	Outpatient	Overall mortality	3 years	9
Joly	2009	France	312	Prospective	Tertiary center	1-year mortality	1 year	9
Parker	2008	USA	223	Retrospective	Outpatient and Inpatient	Overall mortality	2.69 years^a^	6
Joly	2005	France	170	Prospective	Tertiary center	1-year mortality	1 year	9
Rzany	2002	Germany	369	Retrospective	Inpatient	1-year mortality	1.9 years^a^	9
Joly	2002	France	341	Prospective	Tertiary center	1-year mortality	1 year	9
Roujeau	1998	France	217	Retrospective	Tertiary center	6 month mortality	1.5 year^a^	8
Bernard	1997	France	94	Prospective	Tertiary center	1-year mortality	1 year	7

^a^Follow up time in median follow up time

**Table 2:** Quality assessment of included studies

	Lee 2014	Cai 2014	Gual 2014	Li 2013	Zhang 2013	Cortés 2012	Cortés 2011	Joly 2009	Parker 2008	Joly 2005	Rzany 2002	Joly 2002	Roujeau 1998	Bernard 1997
Representativeness of the exposed cohort	1	1	1	1	1	1	1	1	1	1	1	1	1	1
Selection of the non-exposed cohort	1	1	1	1	1	1	1	1	1	1	1	1	1	1
Ascertainment of exposure	1	1	1	1	1	1	1	1	1	1	1	1	1	1
Demonstration that outcome of interest was not present at start of study	1	1	1	1	1	1	1	1	1	1	1	1	1	1
Comparability	2	2	2	2	2	2	2	2	0	2	2	2	2	0
Assessment of outcome	1	1	1	1	1	1	1	1	1	1	1	1	1	1
Was follow-up long enough for outcomes to occur	1	1	1	1	1	1	1	1	1	1	1	1	1	1
Adequacy of follow-up of cohort	0	0	1	0	1	0	1	1	0	1	1	1	0	1
Total score	8	8	9	8	9	8	9	9	6	9	9	9	8	7

### Quantitative synthesis

#### Age

**Fig. 2:**
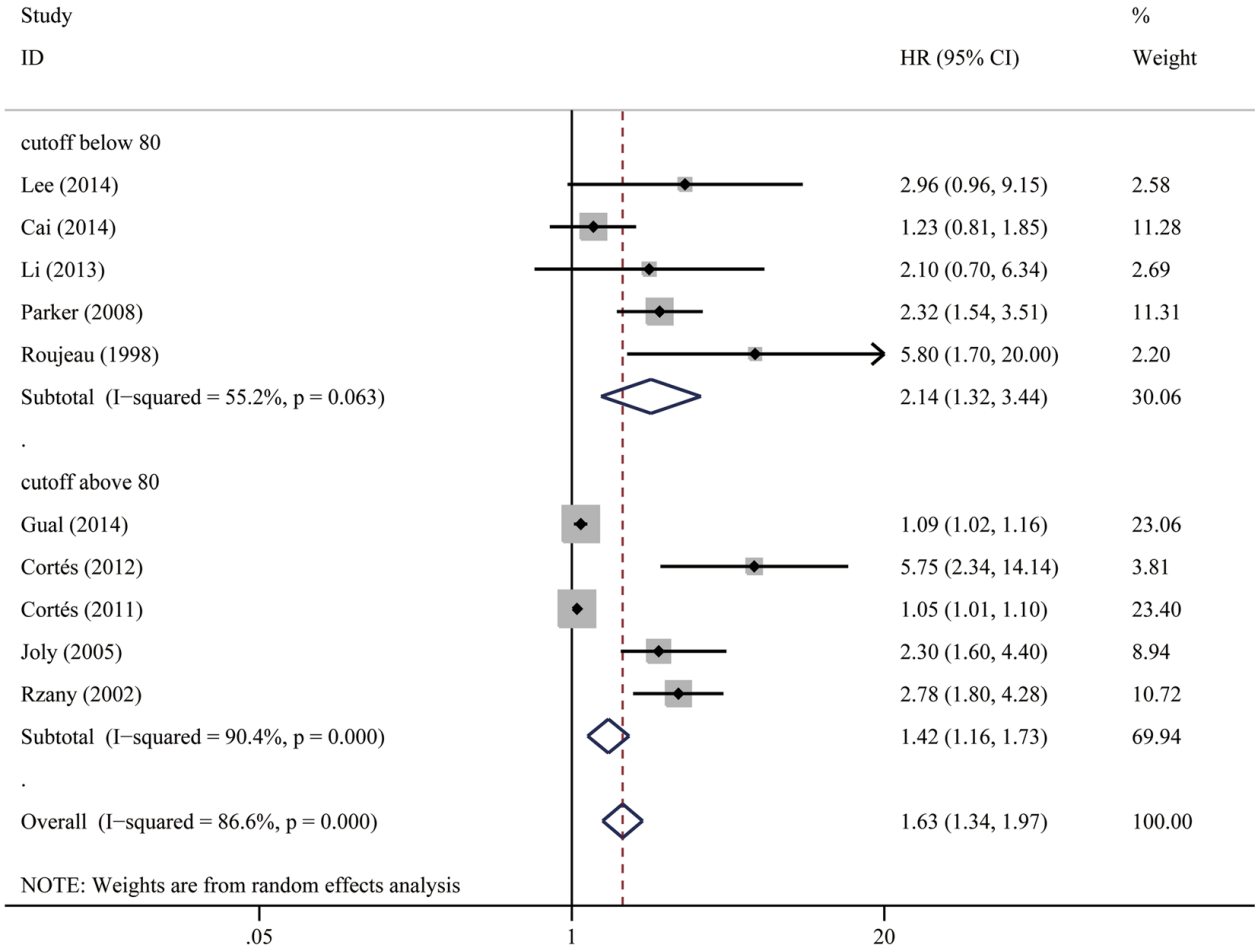
Forest plots: association between bullous pemphigoid mortality and old age

**Fig. 3:**
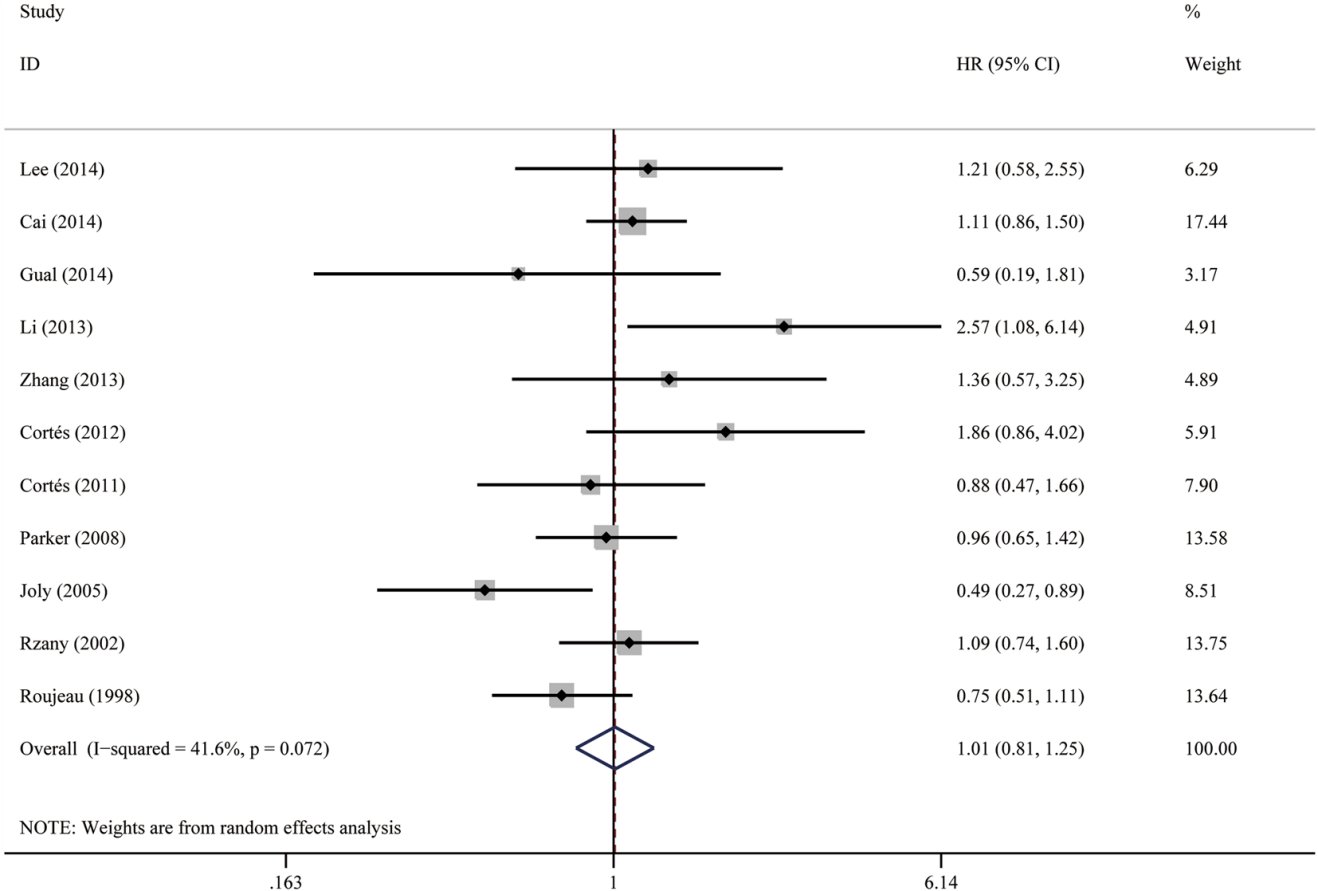
Forest plots: association between bullous pemphigoid mortality and gender

**Fig. 4:**
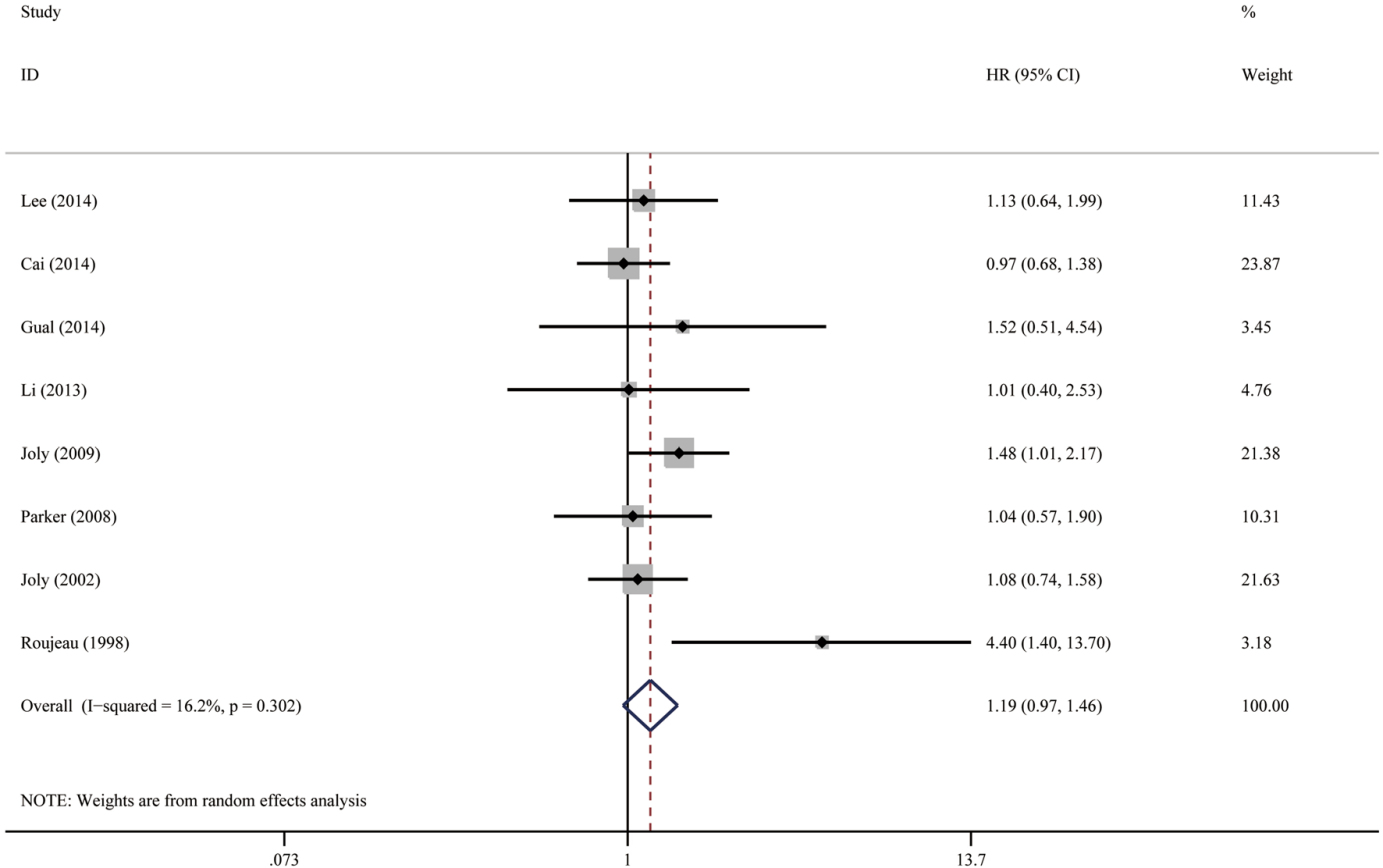
Forest plots: association between bullous pemphigoid mortality and extensive disease

**Fig. 5:**
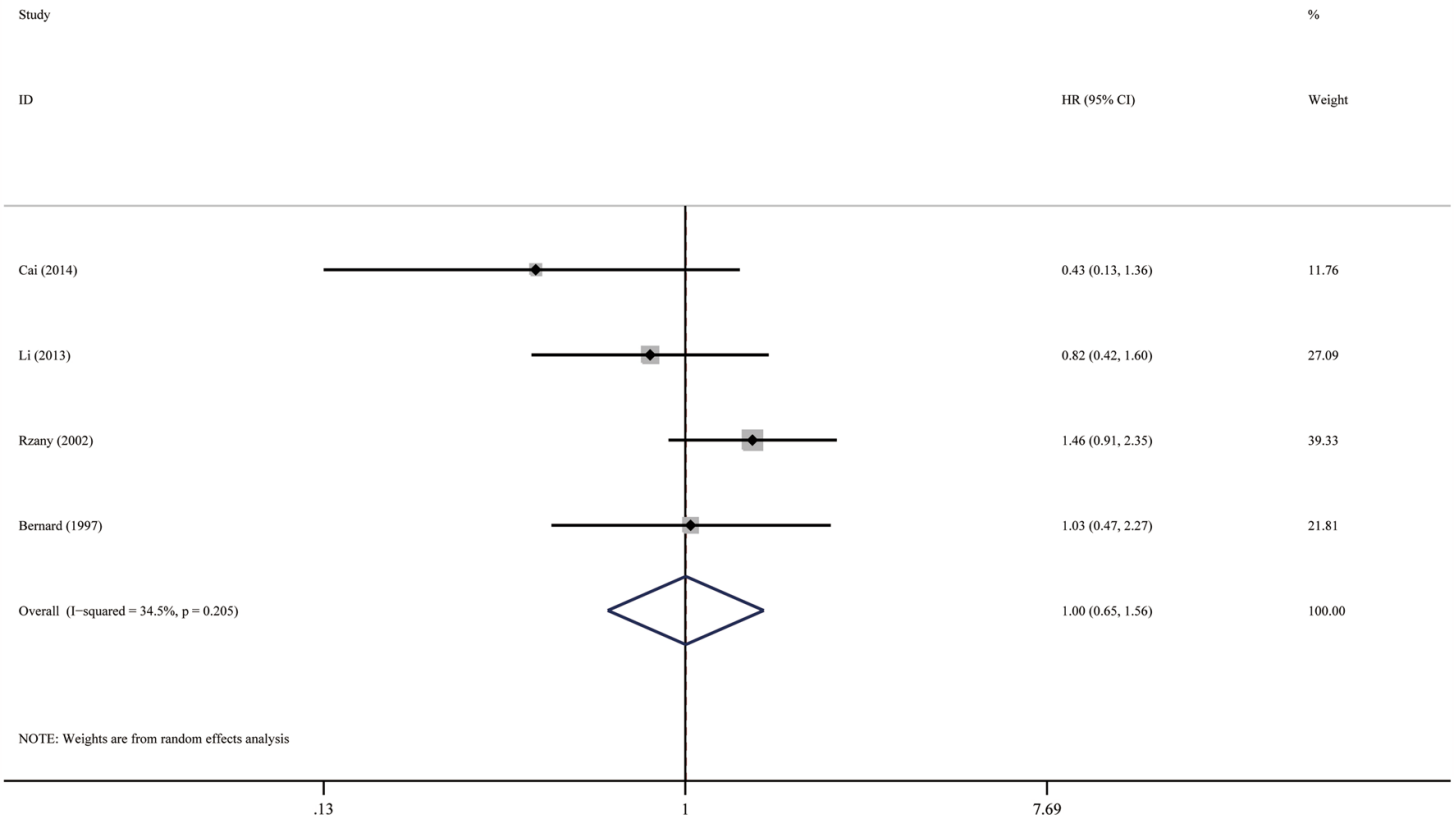
Forest plots: association between bullous pemphigoid mortality and mucosal lesion

**Fig. 6:**
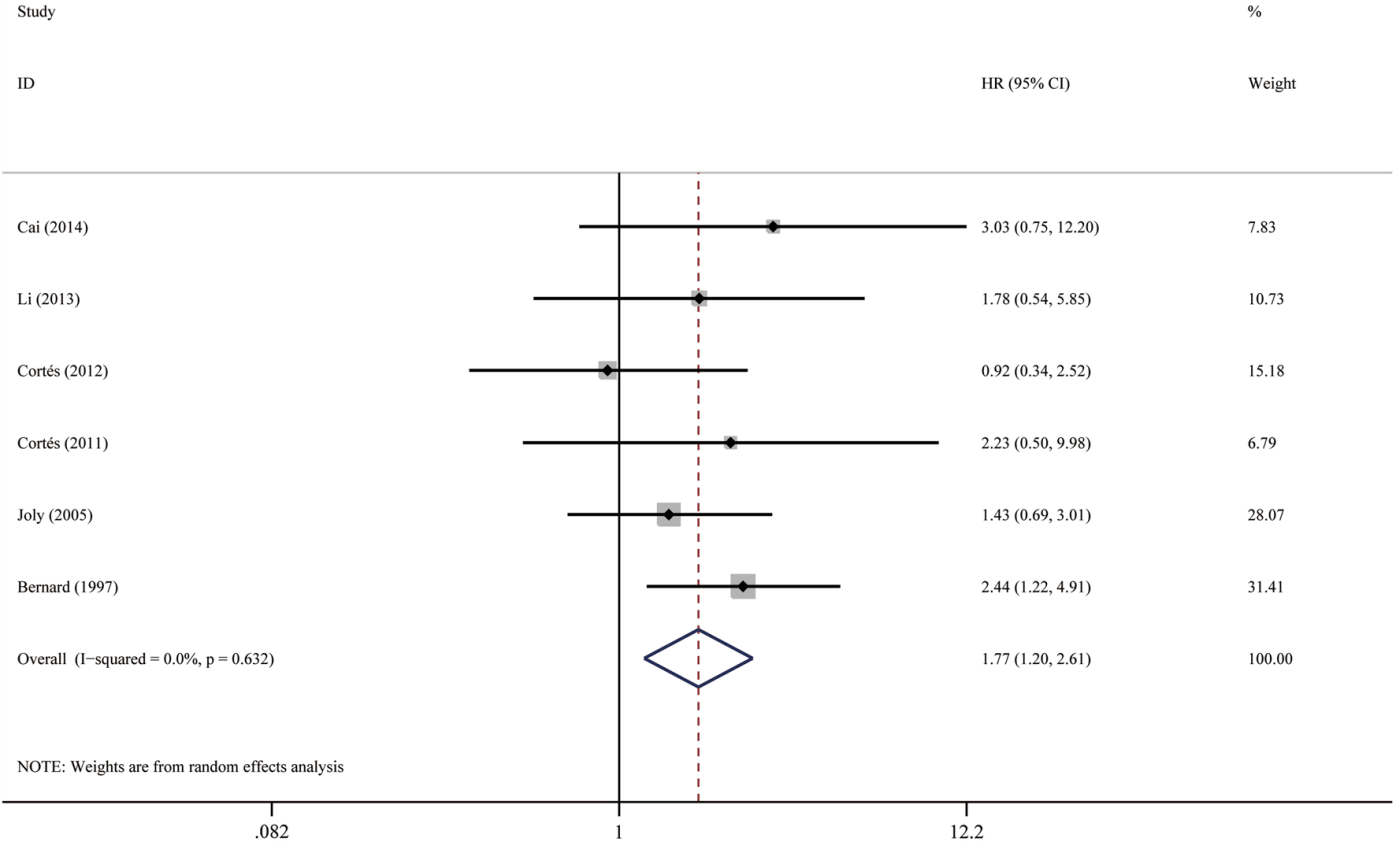
Forest plots: association between bullous pemphigoid mortality and circulating BP autoantibody

**Fig. 7:**
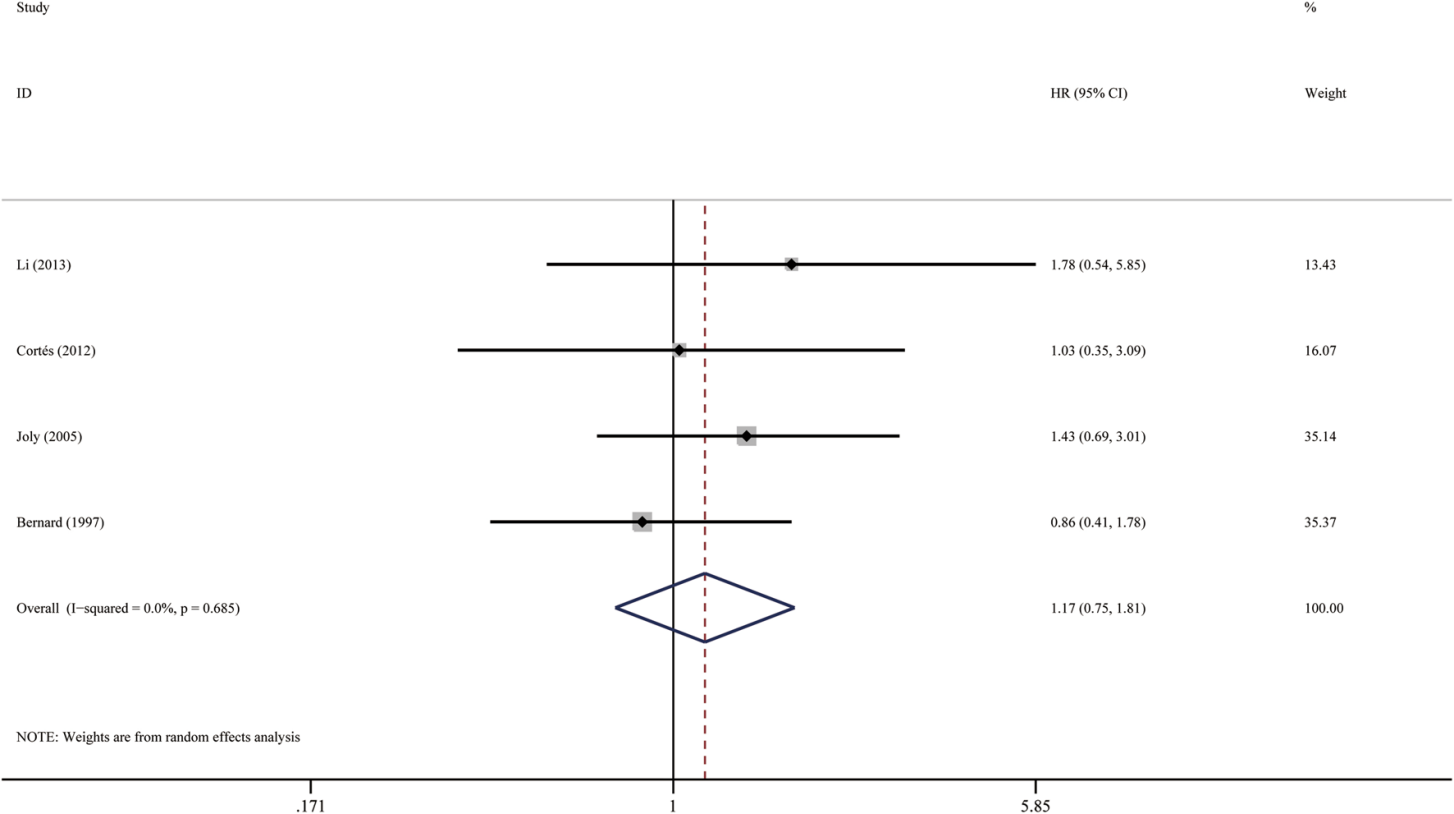
Forest plots: association between bullous pemphigoid mortality and positive IIF

**Fig. 8:**
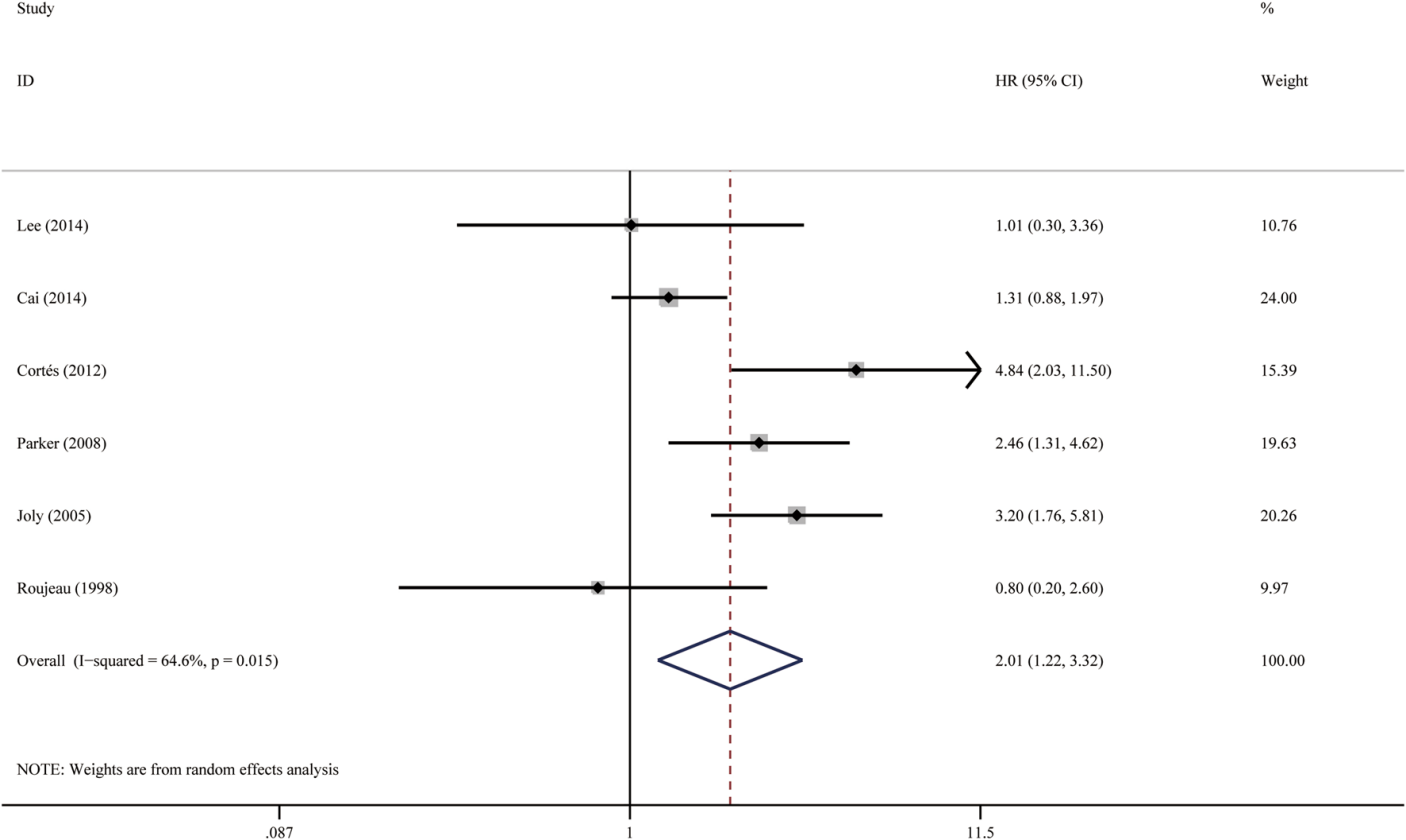
Forest plots: association between bullous pemphigoid mortality and dementia

**Fig. 9:**
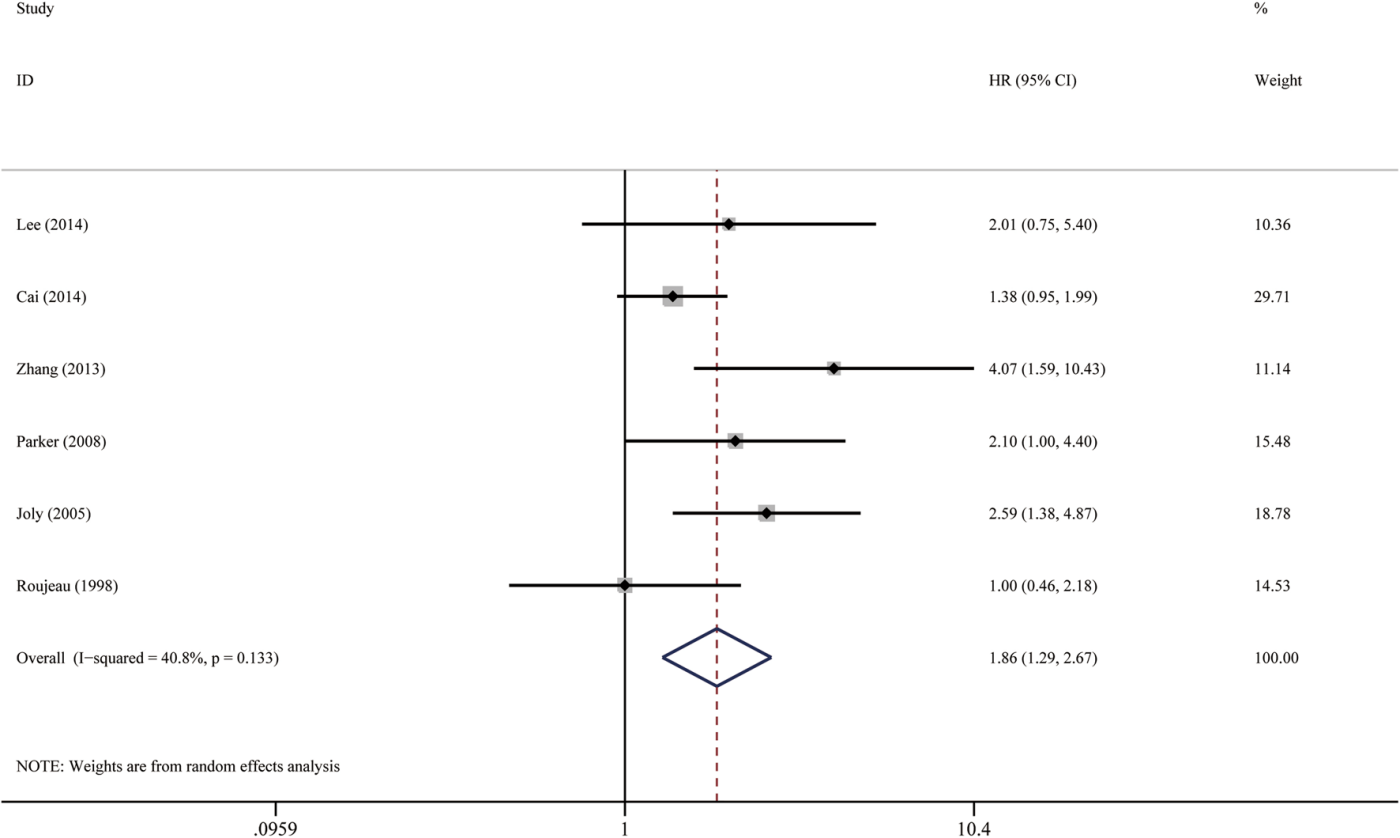
Forest plots: association between bullous pemphigoid mortality and stroke

**Fig. 10:**
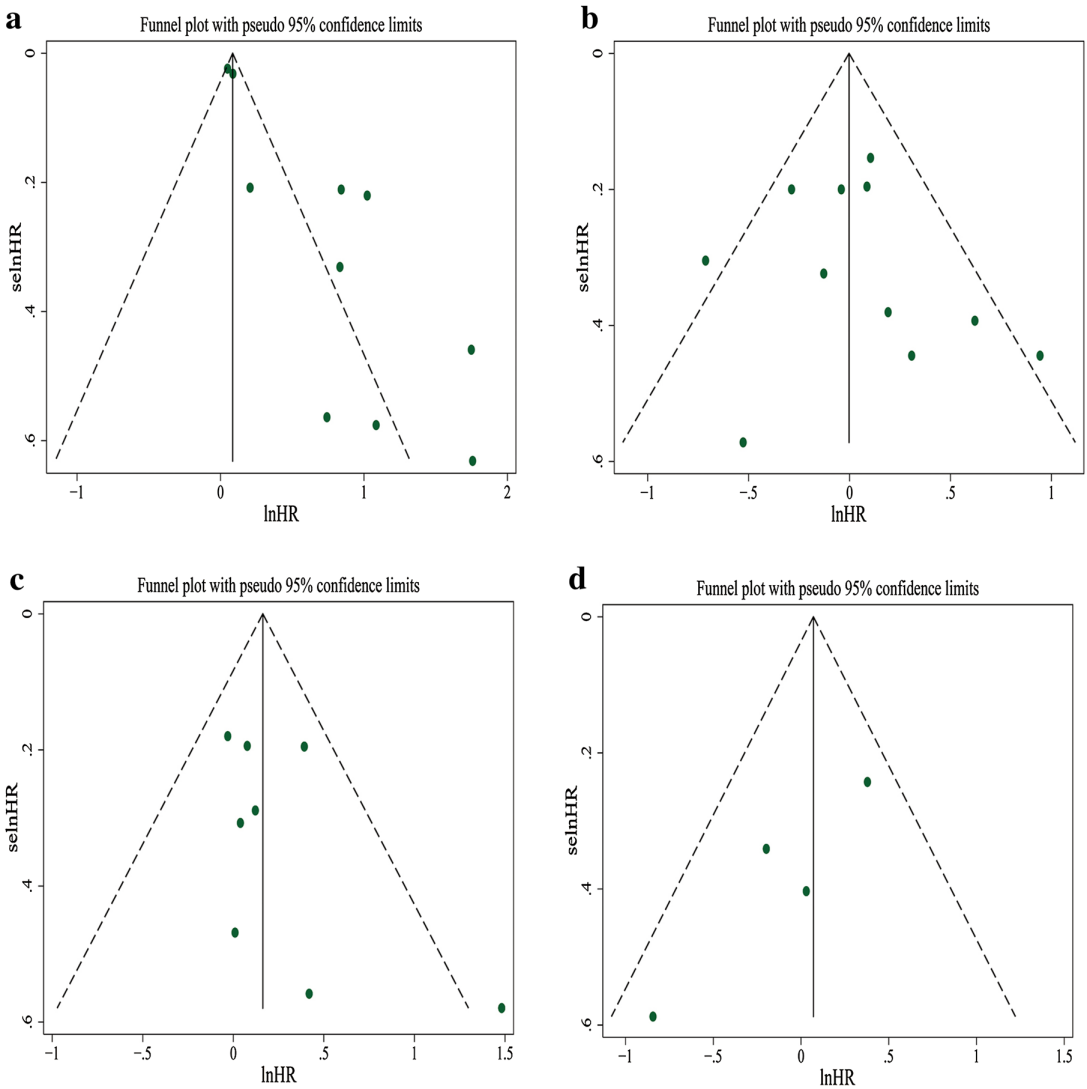

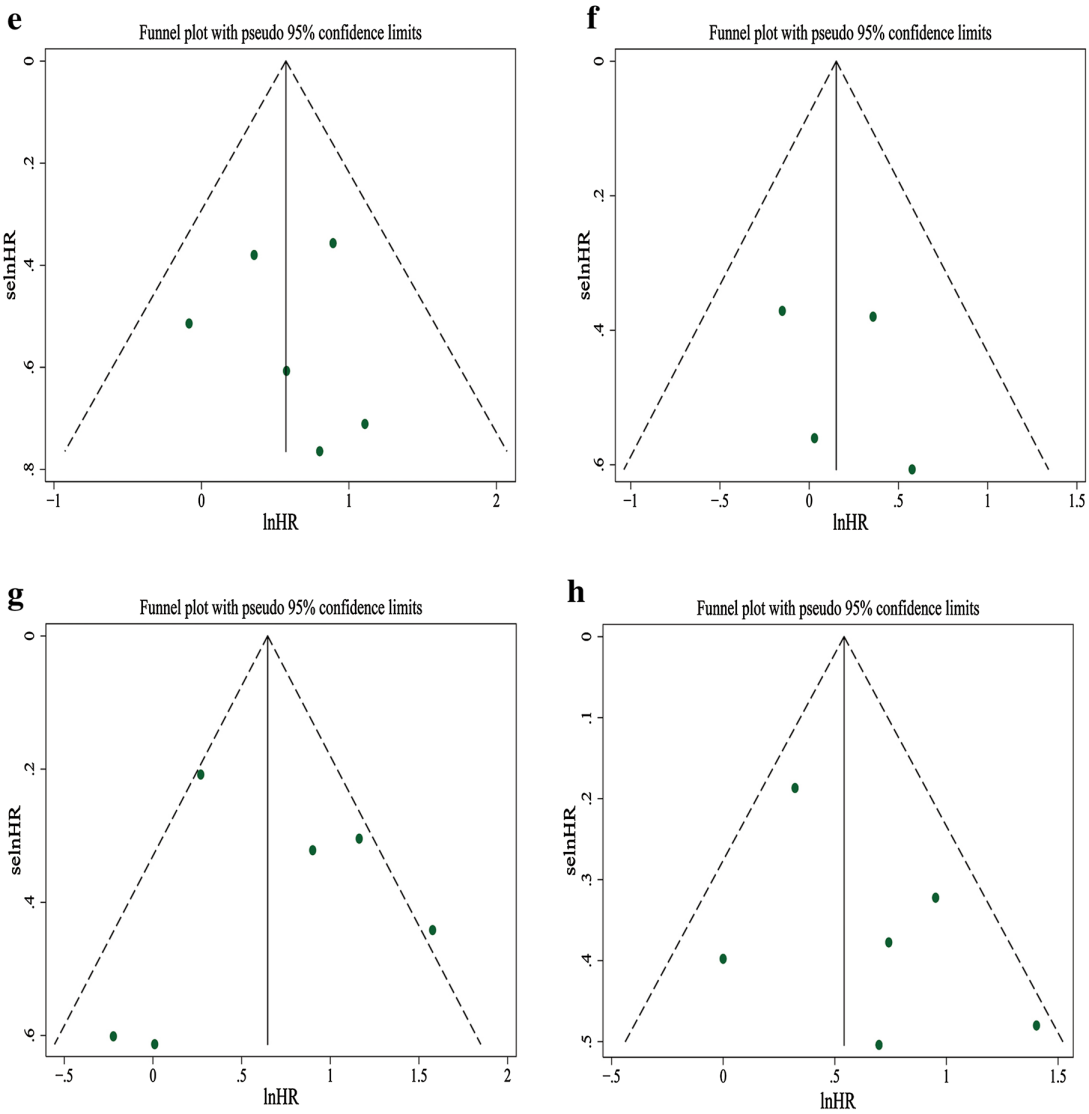
Funnel plots of studies evaluating the relationship between bullous pemphigoid mortality and potential prognostic factors **a** old age, **b** gender (male vs female), **c** extensive disease, **d** mucosal lesion, **e** circulating autoantibody, **f** positive IIF, **g** dementia, **h** stroke

To investigate the effect of age on survival, we included the ten studies which compared survival outcome on elderly BP patients with younger age patient populations. In most studies, patients’ median or mean age at the time of diagnosis was used as the cut-off value to categorize young and elderly patients, and the cut-off value ranged from 67 to 83 years across studies. The pooled HR of old age for mortality risk was 1.63 (95% CI 1.34–1.97, *P* < 0.001). Significant heterogeneity was seen among studies (*I* ^2^ = 86.6%, *P* < 0.001). In subgroup analysis by age cut-off value, studies using cut-off age ≥80 (HR 1.42; 95% CI 1.16–1.73) and studies using cut-off age <80 (HR 2.14; 95% CI 1.32–3.44) both showed statistical significance. Study heterogeneity remained moderate to high for studies using cut-off age ≥80 (*I* ^2^ = 90.4%, *P* < 0.001) and studies using cut-off age <80 (*I* ^2^ = 55.2%, *P* = 0.063). Visual inspection of the funnel plot suggested obvious asymmetry, and this was further supported by Egger’s test showing evidence for reporting bias (*P* = 0.001). “Trim and fill” method showed that five studies had to be added to correct funnel plot asymmetry, and the pooled outcome was no longer statistically significant (HR 1.17; 95% CI 0.96–1.42, *P* = 0.130). Figures 2, 3, 4, 5, 6, 7, 8, 9 show the forest plots with summary measures of the associations between BP mortality and potential prognostic factors. Figure 10 illustrates the funnel plots of component studies in this meta-analysis. Table 3 summarizes the pooled results of potential risk predictors.

**Table 3:** Summary of pooled results of potential prognostic factors

Prognostic factor	Number of studies	Participants	Hazard ratio (95% CI)	Statistical difference
Old age	10	1922	1.63 (1.34–1.97)	S (*P* < 0.001)
Gender (male vs female)	11	2016	1.01 (0.81–1.25)	NS (*P* = 0.944)
Extensive disease	8	1861	1.19 (0.97–1.46)	NS (*P* = 0.102)
Mucosal lesion	4	962	1.00 (0.65–1.56)	NS (*P* = 0.992)
Circulating autoantibody by any detection method	6	938	1.77 (1.20–2.62)	S (*P* = 0.004)
Positive IIF	4	464	1.17 (0.75–1.81)	NS (*P* = 0.488)
Dementia	6	1197	2.01 (1.22–3.33)	S (*P* = 0.006)
Stroke	6	1231	1.86 (1.29–2.67)	S (*P* < 0.001)

*S* significant, *NS* non-significant, *IIF* indirect immunofluorescence

#### Gender

No significant impact was observed for BP mortality, and the pooled HR of male vs female was 1.01 (95% CI 0.81–1.25, *P* = 0.944), as shown in Fig. 3. Low-to-moderate heterogeneity was observed between the studies (*I* ^2^ = 41.6%, *P* = 0.072). Visual inspection of the funnel plot did not reveal obvious asymmetry (Fig. 10), and it was confirmed by Egger’s test (*P* = 0.706).

#### Extensive disease

The definition of disease extent varied among studies. Joly et al. have defined extensive disease as the occurrence of more than ten new blisters per day [16, 17]. Two studies graded BP disease severity based on the percentage of body surface area involvement as follows: mild, <10%; moderate, 10–30%; and severe, >30% [21, 27]. Four studies defined localized disease as isolated lesions in one anatomical region, and generalized disease for patients with moderate-to-diffuse lesions in two or more regions [3, 12, 22, 29]. For simplicity, we combined these eight studies and the pooled HR of extensive disease was 1.19 (95% CI 0.97–1.46, *P* = 0.102), as shown in Fig. 4. No heterogeneity was observed between the studies (*I* ^2^ = 16.2%, *P* = 0.302). Visual inspection of the funnel plot could not rule out publication bias (Fig. 10), but the result of Egger’s test was not significant (*P* = 0.233).

#### Mucosal lesion

The pooled HR of mucosal lesion vs no mucosal lesion was 1.00 (95% CI 0.65–1.56, *P* = 0.992), as illustrated in Fig. 5. The heterogeneity between the studies was low (*I* ^2^ = 34.5%, *P* = 0.205). The funnel plot showed a lack of small size studies with positive association, suggesting probable publication bias (Fig. 10). Egger’s test, however, failed to provide any evidence for small study effect (*P* = 0.079).

#### Circulating BP autoantibody

Because only six studies investigated the relationship between the presence of circulating BP antibodies and patient survival, we pooled the results of all these studies together, regardless of the detection method of antibodies. When a study performed both indirect immunofluorescence (IIF) and enzyme-linked immunosorbent assay (ELISA) or immunoblot, we utilized survival data based on ELISA (preferentially anti-BP180) or immunoblot instead of IIF, since ELISA result was reported to indicate disease activity [10]. The presence of circulating antibodies conferred a 1.77 fold increased risk (95% CI 1.20–2.62, *P* = 0.004), as shown in Fig. 6. The studies were considered homogenous (*I* ^2^ = 0.0%, *P* = 0.632). The funnel plot was roughly symmetrical (Fig. 10). Egger’s test also failed to provide any evidence for small study effect (*P* = 0.859). In addition, we performed analysis for survival results based on positive IIF (HR = 1.17; 95% CI 0.75–1.81, *P* = 0.488), as shown in Fig. 7. The funnel plot was roughly symmetrical (Fig. 10), and Egger’s test was not significant (*P* = 0.622).

#### Dementia

Patients with dementia (including Alzheimer’s dementia and all other types of dementia) were twice more likely to die than patients who did not have dementia. The pooled HR was 2.01 (95% CI 1.22–3.33, *P* = 0.006), as shown in Fig. 8. There was moderate-to-high heterogeneity between the studies (*I* ^2^ = 64.6%, *P* = 0.015). The funnel plot was not perfectly symmetrical (Fig. 10), suggesting possible publication bias. Egger’s test, however, failed to provide any evidence for small study effect (*P* = 0.853).

#### Stroke

Patients with stroke had nearly two-fold increased risk of death compared with patients who did not have this comorbidity. The pooled HR was 1.86 (95% CI 1.29–2.67, *P* < 0.001), as illustrated in Fig. 9. There was low-to-moderate heterogeneity between the studies (*I* ^2^ = 40.8%, *P* = 0.133). Visual inspection of the funnel plot suggested probable reporting bias (Fig. 10), although Egger’s test did not provide any evidence for small study effect (*P* = 0.277).

## Discussion

In this meta-analysis of 14 studies that included 2499 subjects, we found that older age, presence of circulating antibodies regardless of detection method, co-existing dementia, and stroke were associated with an increased risk of BP mortality. No significant association was observed for gender, disease extent, mucosal involvement, or IIF result and survival.

In consistence with most studies included in the current analysis, we found no survival difference based on sex. However, age at diagnosis was associated with increased risk of death, with advanced age being a poor prognostic factor. Stratification of studies based on age cut-off value demonstrated that the effect size was more significant when the cutoff was lower, suggesting that the truly younger patients may have a better prognosis. Discrepancies in the age cut-off values, patient baseline characteristics, and treatment approaches between studies might contribute to the high level of heterogeneity. The pooled result did not remain comparable after “trim and fill” adjustment, suggesting that publication bias might have led to an overestimation of the effect of age on survival. However, the asymmetry in funnel plot may also simply be the play of chance, since only ten studies were included, or true heterogeneity existed because cut-off age varied greatly. Of note, the two outlier investigations by Roujeau et al. [29] and Cortés et al. [5] were small studies with less than 100 participants. Nevertheless, the above finding indicates that younger age at the diagnosis of BP does not necessarily denote a lower risk of mortality, and physicians should be more cautious about patients’ general health condition and comorbidity profile.

As reported previously, more extensive form of BP did not significantly increase the risk on mortality. Although the extent and distribution of disease are a variable susceptible to high variability between centers and may introduce information bias, especially in retrospective studies, heterogeneity between the included studies was low. The presence of mucosal lesion was also not predictive of poor survival outcome in our analysis. Of the other factors related to BP activity that were not included in this meta-analysis due to insufficient data for statistical pooling, such as pruritus [15, 29] and eosinophilia [1, 15, 22, 29], no mortality difference was found by previous investigations. These findings suggest that inherent factors such as age and gender, as well as disease severity at the time of diagnosis, may not influence mortality as much as general condition and concomitant morbidities.

Our analysis corroborated previous finding that IIF is not a sensitive indicator of BP clinical activity [18], but suggested that the presence of circulating antibodies regardless of detection method increased mortality risk, pointing to a predictive role of ELISA test. Unfortunately, since only three studies investigated survival prognosis based on ELISA result, we did not perform statistical pooling for this. To date, IIF plays a key role in the detection of circulating antibodies and is often used as the routine test. However, the circulating autoantibodies can also be detected by more accurate serological assays, including immunoblotting and ELISA [18]. Of note, ELISA has been demonstrated as a useful tool to detect circulating antibodies in BP with its high sensitivity and specificity, particularly to recombinant BP180 antigen [18]. Moreover, it has the ability to reflect the severity of bullous pemphigoid, presumably because autoantibody against BP180 is believed to be pathogenic in BP [10]. It is noteworthy that this represents an intrinsic risk factor that correlates with BP mortality.

Severe concomitant morbidities at the time of diagnosis of bullous pemphigoid, such as neurological disorders, cardiac insufficiency, diabetes, and malignancies, increase the risk of mortality [3, 12, 21]. Interestingly, there is a strong link between BP and neurological diseases. In this study, we evaluated the impact of two most common neurological diseases in BP patients, dementia, and stroke, on the survival of BP patients. Our pooled data indicated a roughly two-fold higher mortality risk for BP patients with dementia or stroke. The exact mechanism underlying the link between neurological diseases and BP has not yet been fully elucidated, but a putative autoimmune reaction against BP antigens in the brain, via a compromised blood–brain barrier, that cross react with the skin have been speculated as a possible cause [20]. Neurological diseases may be responsible for the poor prognosis of some patients with BP because of their altered general condition. However, patients with neurodegenerative disorders are known to have increased blood–brain barrier permeability that may facilitate the generation and crossing of BP autoantibodies into the periphery, worsening skin damage, and clinical outcome [8]. A recent study revealed that in a group of patients with Alzheimer’s disease but no bullous pemphigoid, their sera contained much higher level of BP180 autoantibody than neurologically healthy controls. Furthermore, increased levels of the autoantibody were associated with more severe dementia [19]. Thus, it remains unclear whether neurological disease itself could increase the mortality risk, or the increased risk is conferred by functional impairment and decreased autonomy resulting from neurological disease. Future prospective studies should assess patient’s functional status at initial presentation and employ a multivariate analysis to answer this question.

Meta-analysis of some other prognostic factors was not conducted, because the data were too sparse or heterogeneous to combine analytically. Poor general condition, low karnofsky scale, or being bedridden has been repeatedly reported to be associated with unfavorable outcome [3, 15, 32], with the exception of one study conducted by Lee et al. [21], which did not find a significant association between karnofsky scale and BP mortality after multivariate analysis. Low serum albumin level and high erythrocyte sedimentation rate (ESR) were found to be associated with elevated mortality risk in BP [22, 30]. High ESR may be a surrogate marker for more severe inflammatory status and more severe disease. Serum albumin level is related to patient’s nutritional condition, or underlying diseases, or extensive cutaneous erosions. However, these two factors are easily influenced by various factors, such as other acute and chronic illnesses, so they do not represent intrinsic biological variables of bullous pemphigoid.

Steroid is the first line therapy in both localized and generalized BP [17]. High dosage of systemic corticosteroid or immunosuppressive agents correlates with higher mortality and increased side effects especially in patients at risk, as early deaths in BP patients are mainly of infectious or cardiovascular origin [16]. However, we were not in an optimal situation to validly investigate this issue, because (1) treatment modalities had not been predetermined and controlled in most studies in our analysis; (2) the average dosage of steroid used in different studies varied greatly; (3) dosage and route of administration were largely determined by patients’ disease status; and (4) patients were often administered a combination of systemic steroid, topical steroid, and adjunctive immunomodulators.

Potential limitations need to be considered when interpreting the results of our meta-analysis. First, since most included studies were retrospective and conducted in referral centers, a potential unidentified confounding, information, and selection bias may exist, causing us to interpret our results with caution. Second, the follow-up time varied from 1 to 5 years among studies, which may influence the comparability of the results between component studies. Third, pooled results for some factors, such as mucosal involvement and positive IIF, were based on a limited number of studies. Therefore, we cannot rule out the possibility that insufficient statistical power is present. Finally, we could not evaluate all of the predictive factors for BP mortality in this meta-analysis.

## Conclusion

Taken together, our results indicated that BP patients with older age, circulating BP autoantibodies, dementia, and stroke are at greater risk of mortality. Given that this conclusion was made using pooled data derived from multiple studies, such information has value for the purposes of patient education and prognosticating risk during the treatment process. Prognostic studies based on prospective design have the advantage of ruling out confounding effect, such as treatment regimen. More rigorously designed prospective studies with long-term follow-up duration are still required for future supplements and updates.

## Electronic supplementary material

Below is the link to the electronic supplementary material.

Supplementary material 1 (PDF 3351 KB)
