# Supplementary material for: Prognostic factors for mortality in patients with bullous pemphigoid: a meta-analysis
Source: Arch Dermatol Res. 2017 Mar 19;309(5):335–47. doi: 10.1007/s00403-017-1736-1 (PMC5486568; doi:10.1007/s00403-017-1736-1)
Supplement: Supplementary file 1 — Supplementary material 1 (PDF 3351 KB) [file 403_2017_1736_MOESM1_ESM.pdf]

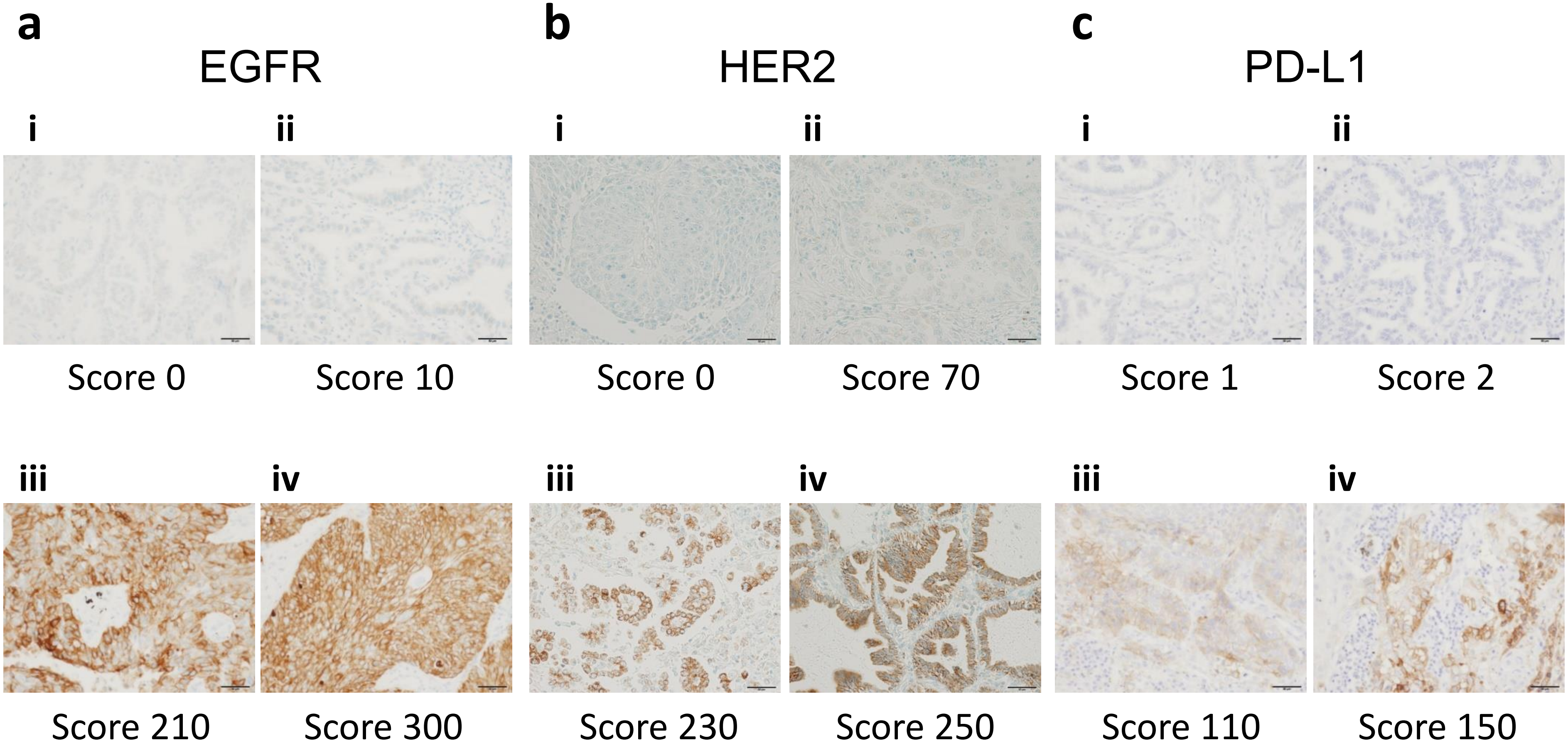

Supplementary Figure 1

Immunohistochemical staining of EGFR, HER2, and PD-L1 in NSCLC tissues. Representative staining for (**a**) EGFR, (**b**) HER2, and (**c**) PD-L1 in tumor tissues (×100 magnification). The panels show images corresponding to different intensity scores of EGFR, HER2, and PD-L1 expression. (**i, ii**): low and (**iii, iv**): high. Bar: 50 μm.

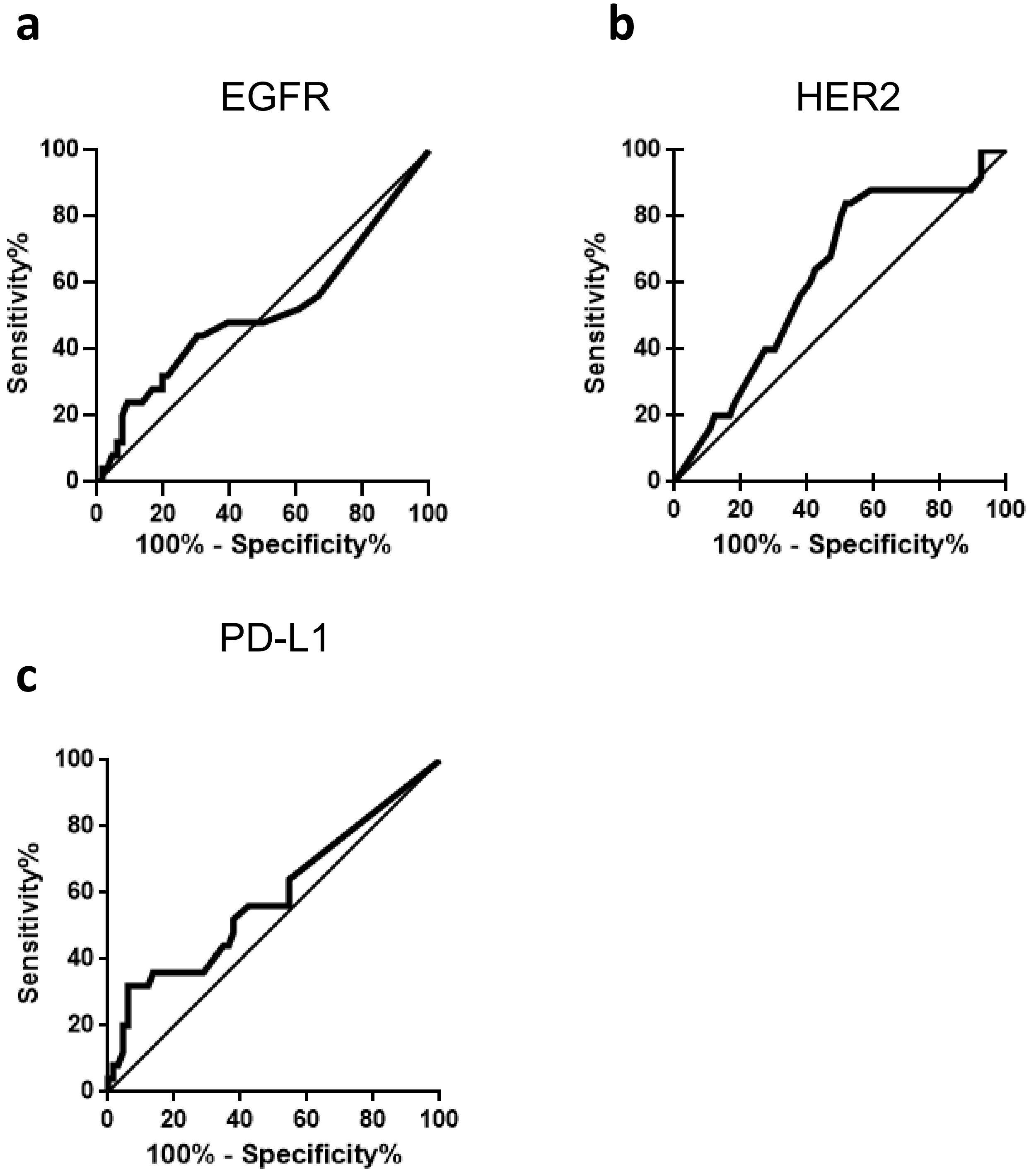

Supplementary Figure 2

Receiver operating characteristics (ROC) curve for predicting recurrence free survival (RFS). RFS-EGFR: AUC 0.51 (95 % CI 0.37–0.66). RFS-HER2: AUC 0.63 (95 % CI 0.50–0.75). RFS-PD-L1: AUC 0.59 (95 % CI 0.45–0.73).

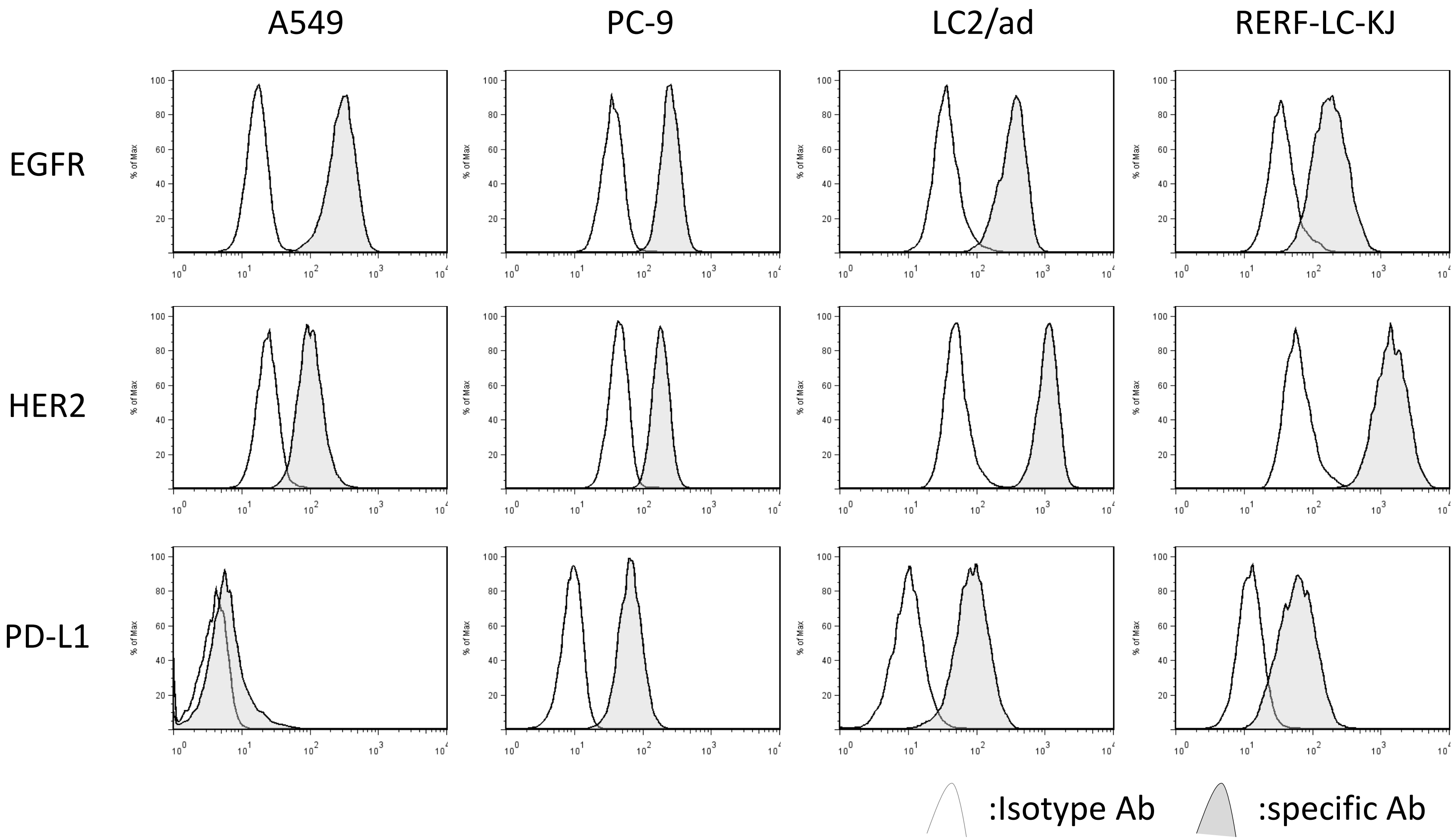

Supplementary Figure 3

Basal expression of EGFR, HER2, and PD-L1 in 4 NSCLC cell lines. The expression levels of each molecule were evaluated by flow cytometry.

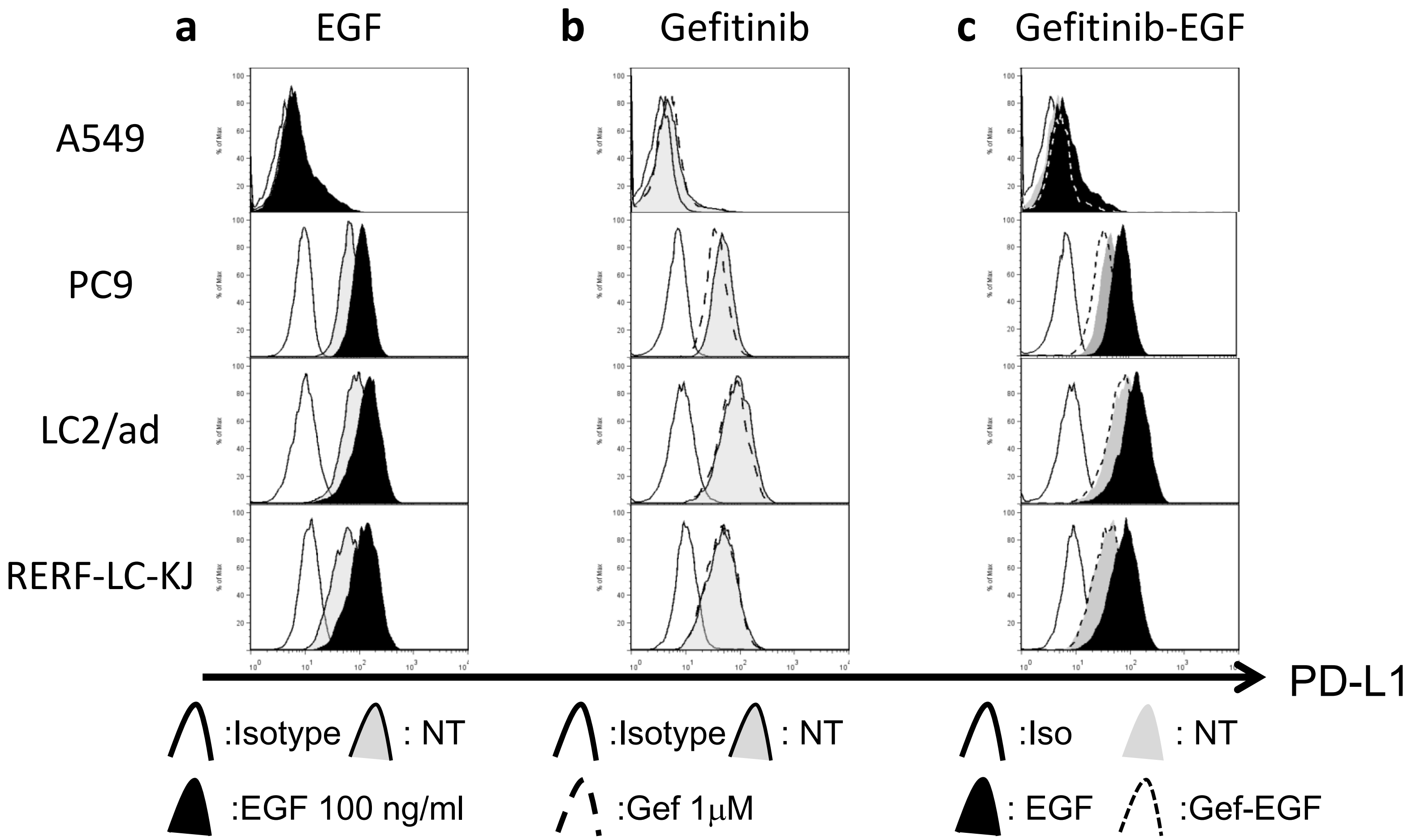

Supplementary Figure 4. **PD-L1 expression is upregulated by EGF, and EGF-induced PD-L1 is blocked by EGFR tyrosine kinase inhibitor gefitinib in NSCLC cell lines.** (**a**) Four NSCLC cell lines were treated with 100 ng/mL of EGF or (**b**) 1 μM of gefitinib for 24 h, then the expression levels of PD-L1 were individually assessed using flow cytometry. (**c**) Four NSCLC cell lines were pretreated with 1 μM of gefitinib for 2 h followed by 100 ng/mL of EGF for 24 h, then the expression levels of PD-L1 were individually assessed using flow cytometry. Representative histograms from 3 independent experiments are shown.

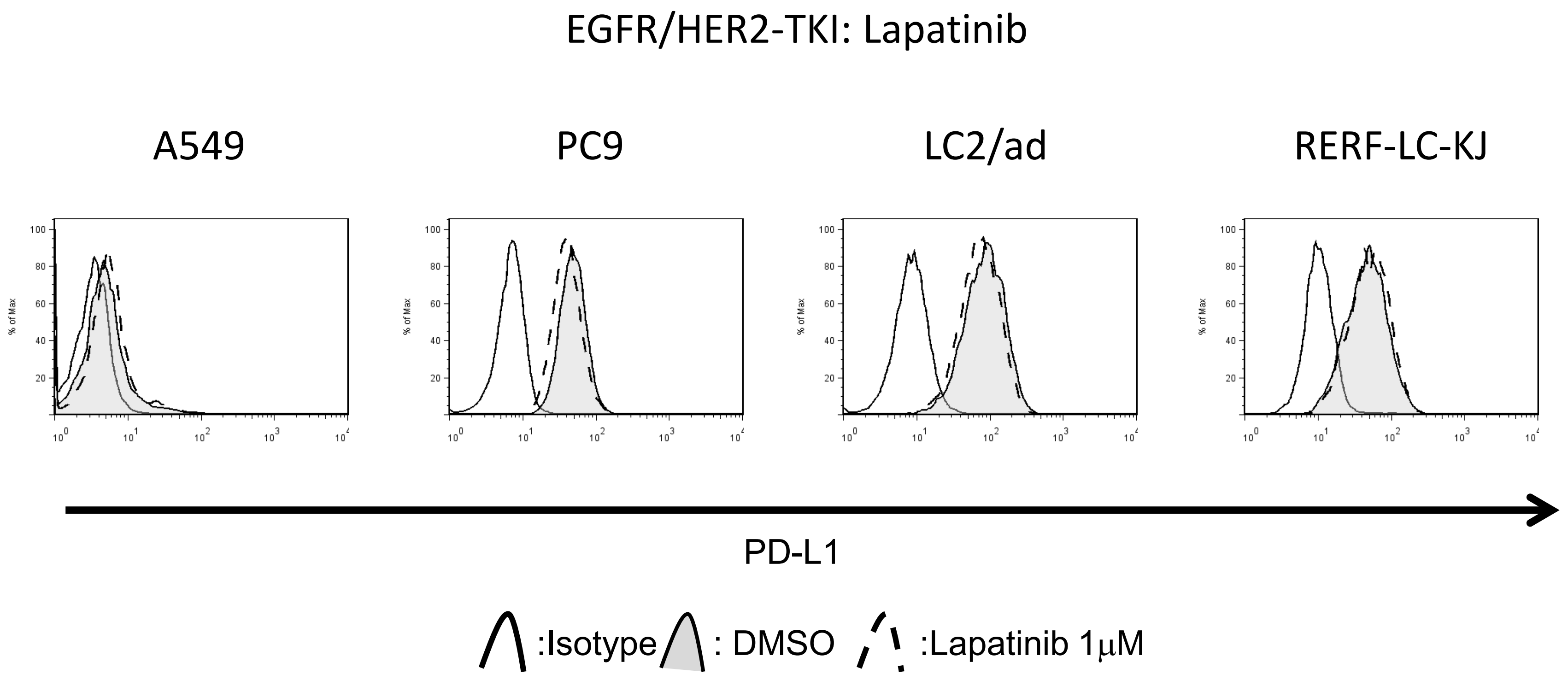

Supplementary Figure 5

Expression of PD-L1 in NSCLC cells treated with EGFR/HER2 dual tyrosine kinase inhibitor lapatinib. Four NSCLC cell lines were treated with 1 μM of lapatinib for 24 h, then the expression levels of PD-L1 were individually assessed using flow cytometry. Representative histograms from 3 independent experiments are shown.

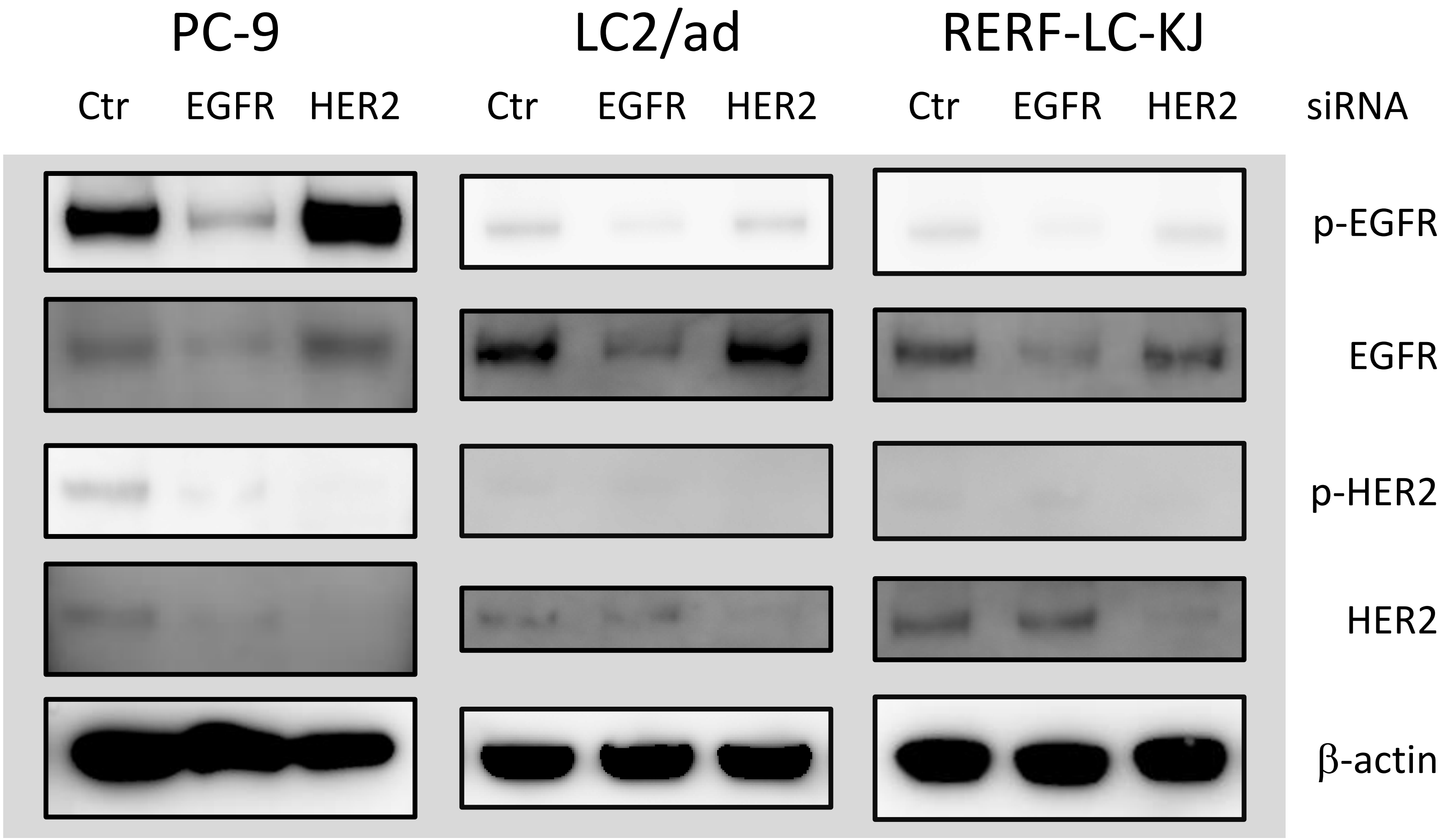

Supplementary Figure 6

Immunoblotting assay to assess the efficacy of small interfering RNA assay in NSCLC cells. PD-L1 high-expressing PC-9, LC2/ad, and RERF-LC-KJ cells were transfected with EGFR, HER2, or control siRNA for 48 h. The expression levels of both receptors and β-actin were assessed using western blot analyses.
